# *Streptococcus pneumoniae* NanC: STRUCTURAL INSIGHTS INTO THE SPECIFICITY AND MECHANISM OF A SIALIDASE THAT PRODUCES A SIALIDASE INHIBITOR*

**DOI:** 10.1074/jbc.M115.673632

**Published:** 2015-09-14

**Authors:** C. David Owen, Petra Lukacik, Jane A. Potter, Olivia Sleator, Garry L. Taylor, Martin A. Walsh

**Affiliations:** From the ‡ Biomedical Sciences Research Complex, University of St. Andrews, St. Andrews, Fife KY16 9ST, United Kingdom,; § Diamond Light Source and; ¶ Research Complex at Harwell, Harwell Science and Innovation Campus, Didcot, Oxfordshire OX11 0FA, United Kingdom, and; the ‖ Medical Research Council France, c/o European Synchrotron Radiation Facility, BP 220, 38043 Grenoble, France

**Keywords:** glycoside hydrolase, neuraminidase, sialic acid, sialidase, Streptococcus, NanC, carbohydrate-binding module, neu5ac2en, pneumococcus

## Abstract

*Streptococcus pneumoniae* is an important human pathogen that causes a range of disease states. Sialidases are important bacterial virulence factors. There are three pneumococcal sialidases: NanA, NanB, and NanC. NanC is an unusual sialidase in that its primary reaction product is 2-deoxy-2,3-didehydro-*N*-acetylneuraminic acid (Neu5Ac2en, also known as DANA), a nonspecific hydrolytic sialidase inhibitor. The production of Neu5Ac2en from α2–3-linked sialosides by the catalytic domain is confirmed within a crystal structure. A covalent complex with 3-fluoro-β-*N*-acetylneuraminic acid is also presented, suggesting a common mechanism with other sialidases up to the final step of product formation. A conformation change in an active site hydrophobic loop on ligand binding constricts the entrance to the active site. In addition, the distance between the catalytic acid/base (Asp-315) and the ligand anomeric carbon is unusually short. These features facilitate a novel sialidase reaction in which the final step of product formation is direct abstraction of the C3 proton by the active site aspartic acid, forming Neu5Ac2en. NanC also possesses a carbohydrate-binding module, which is shown to bind α2–3- and α2–6-linked sialosides, as well as *N*-acetylneuraminic acid, which is captured in the crystal structure following hydration of Neu5Ac2en by NanC. Overall, the pneumococcal sialidases show remarkable mechanistic diversity while maintaining a common structural scaffold.

## Teaser

**Background:** The *Streptococcus pneumoniae* sialidase NanC produces a nonspecific inhibitor of hydrolytic sialidases.

**Results:** The NanC crystal structure is presented in complex with mechanistically relevant ligands.

**Conclusion:** A constricted and hydrophobic active site produces 2-deoxy-2,3-didehydro-*N*-acetylneuraminic acid (Neu5Ac2en, also known as DANA) via a covalent intermediate and direct proton abstraction by a catalytic aspartic acid.

**Significance:** Insights into an unusual reaction mechanism will aid the design of sialidase inhibitors.

## Introduction

The Gram-positive bacterium *Streptococcus pneumoniae* is a common resident of the human nasopharynx. Although carriage is normally asymptomatic, the bacterium can cause severe illness, particularly in the very young, the elderly, and the immunocompromised, and can be triggered following infection with the influenza virus (1, 2). *S. pneumoniae* is a major cause of bacterial meningitis (3), the most frequent cause of otitis media and sepsis in children, and the primary cause of community-acquired and hospital-acquired pneumonia in adults (4). Conjugate vaccines have successfully targeted the most prevalent strains (5, 6). However, the disease burden rebounds because of replacement disease caused by strains not included in the vaccine (7–9). Furthermore, the search for novel therapeutics against *S. pneumoniae* is becoming more pertinent in view of decreasing antibiotic efficacy because of increased prevalence of single and multidrug-resistant strains (10, 11).

Pneumococcal sialidases are implicated in host colonization and pathogenesis (12). They facilitate invasion through desialylation of the host cell surface decrypting sites for bacterial adhesion (13) and promote biofilm formation (14). The carbohydrate-binding module (CBM)^5^ associated with the NanA sialidase has been shown to be involved in cell surface binding and invasion of the blood-brain barrier (15). Finally, the release of sialic acid from host cells by pneumococcal sialidases has also been proposed as an important source of both carbon and energy (16).

Sialidases show promise as targets for both vaccines and small molecule inhibitors (17–19). The latter strategy has already been adopted and proven successful against the influenza virus (20). *S. pneumoniae* produces up to three distinct sialidase enzymes: NanA, NanB, and NanC. Among 342 pneumococcal strains, the *nanA*, *nanB*, and *nanC* genes were present in 100, 96, and 51% of isolates, respectively, with significantly higher *nanC* prevalence in cerebrospinal fluid isolates in comparison with carriage isolates from the upper respiratory tract (21). More recently, the *nanC* gene was found to have an increased occurrence in isolates from children with haemolytic-uraemic syndrome compared with non-haemolytic uraemic syndrome controls (22).

The crystal structures for NanA and NanB are available and show domain conservation: a signal sequence, an N-terminal carbohydrate-binding module, and a catalytic β-propeller domain with an irregular inserted (I) domain of unknown function that protrudes from the catalytic domain (23–26). NanC shares 25% sequence identity with NanA and 51% sequence identity with NanB. NanB and NanC are of similar size, 78 and 82 kDa, respectively. NanA is largest at 115 kDa because of the presence of a C-terminal membrane-anchoring domain.

The pneumococcal sialidases vary in substrate specificities, mechanism, and kinetic parameters. Although NanA can cleave a range of sialic acid substrates including those with α2–3, α2–6, and α2–8 glycosidic linkages (23, 27), NanB and NanC have specificity for α2–3-linked substrates (24, 25, 27). NanA is a typical exo or hydrolytic sialidase, hydrolyzing its substrates to release *N*-acetylneuraminic acid (Neu5Ac). NanB is an intramolecular *trans*-sialidase generating 2,7-anhydro-Neu5Ac as a reaction product (24, 25). Despite the high sequence similarity to NanB, NanC has been shown to differ in that it initially generates Neu5Ac2en, a molecule that inhibits NanA with a *K_i_* of 2 μm (24, 28). In fact, Neu5Ac2en appears to be a nonspecific inhibitor of hydrolytic sialidases with micromolar inhibition also observed against influenza virus neuraminidase (29), the human sialidases (30, 31), and VcNA from *Vibrio cholerae* (32). Neu5Ac2en is a very poor inhibitor of NanC with a *K_i_* of 2–3 mm (27, 28). Following depletion of the α2–3-linked substrate, NanC can hydrate Neu5Ac2en to Neu5Ac (27). Here we present the crystal structure of NanC and its catalytic active site complexes with the reaction product Neu5Ac2en, a covalently bound reaction intermediate, 3-fluoro-β-*N*-acetylneuraminic acid (3F-β-Neu5Ac), and the viral neuraminidase inhibitor oseltamivir carboxylate (OC). In addition, complexes with the substrate α2–3-sialyllactose (3′SL), α2–6-sialyllactose (6′SL), and Neu5Ac in the CBM binding site of the catalytic domain are described. Together, these data provide a structural basis for the unusual production of Neu5Ac2en by this enzyme.

## Experimental Procedures

Coordinates for all crystal structures have been deposited in the Protein Data Bank. Two separate cloning, expression, and purification schemes were used. PDB codes 4YW0, 4YW1, 4YW2, 4YW3, and 4YW5 were obtained from scheme A, and PDB codes 4YZ1, 4YZ2, 4YZ4, and 4YZ5 were obtained from scheme B as described below. Differences between the two schemes will be highlighted and referred to as A or B throughout the methods description.

### 

#### 

##### Cloning

For A, the *S. pneumoniae* TIGR4 *nanC* gene was expressed in the pet21b vector (28). The construct encompassed residues 28–740. For B, genomic DNA purified from *S. pneumoniae* TIGR4 (kindly supplied by Dr. Samantha King, Nationwide Children's Hospital, Columbus, Ohio) was used as a template for PCR amplification of the NanC protein-coding region. The In-Fusion® cloning method was used to insert the PCR product into a pOPINF plasmid vector (33). The final construct encompassed residues 83–740.

##### Expression and Purification

Recombinant plasmids (A and B) were expressed in *Escherichia coli* Rosetta (DE3) expression strain (Novagen) in autoinduction media (A, Formedium; B, Overnight Express TB autoinduction media from Novagen). The cultures were initially grown at 37 °C, followed by prolonged growth at lowered temperatures (A, 200 rpm, 60 h, 16 °C; B, 230 rpm, 20 h, 25 °C). The cells were harvested by centrifugation, resuspended in lysis buffer (A, 20 mm Tris-HCl, pH 7.5, 50 mm NaCl, 5 mm imidazole; B, 50 mm Tris, pH 7.5, 500 mm NaCl, 30 mm imidazole, pH 7.5, 0.2% Tween 20, 5% w/v glycerol) supplemented with DNase I (10–20 μg/ml) and Complete protease inhibitor mixture tablets (Roche), and lysed using a constant flow cell disrupter. Insoluble components were removed by centrifugation. Soluble lysate was loaded onto nickel-Sepharose columns (GE Healthcare) equilibrated in (B, Tween 20-free) lysis buffer. Bound protein was washed extensively using lysis buffer (A, containing 20 mm imidazole; B, excluding Tween 20) and eluted using an imidazole step gradient (A, lysis buffer containing 60 mm imidazole; B, lysis buffer containing 500 mm imidazole and excluding Tween 20).

In scheme A, the eluted protein fraction was diluted 1:2 with 20 mm Tris-HCl, pH 7.5, and loaded onto a 5-ml SP-FF cation-exchange column (GE Healthcare). The bound protein was washed using 20 mm Tris-HCl, pH 7.5, 100 mm NaCl and eluted using 20 mm Tris-HCl, 300 mm NaCl. The major peak from (A, cation-exchange; B, nickel affinity purification) was concentrated and subjected to size exclusion chromatography using a 120-ml Sephacryl S-200 column (GE Healthcare) equilibrated in 20 mm Tris, pH 7.5 (A) or 20 mm Tris, pH 7.5, 200 mm NaCl, 5% glycerol (B). In scheme B, post size exclusion, the protein was subjected to His_6_ tag cleavage by incubation with 0.1% (w/w) human rhinovirus B 3C protease (14 h, 4 °C), followed by reverse His tag purification.

##### Protein Crystallization

Promising crystals were obtained from 16% PEG8000, 20% glycerol, 40 mm KH_2_PO_4_ at 20 mg/ml (A) and 20% PEG3350, 0.25 m ammonium sulfate, 0.1 m Hepes, pH 8.0, at 10 mg/ml (B). These initial crystals were optimized by varying crystallization droplet size and protein to precipitant ratio (A, 1 μl 7.5 mg/ml protein, 2 μl of reservoir solution, 0.5 μl of seed stock; B, 0.5 μl of 10 mg/ml protein, 0.5 μl of reservoir), incorporation of additives (A, 10% degassed sugar-free Irn-Bru (A.G. Barr); B, 7.5% ethanol or isopropanol), and microseeding (A) (34). Additives were critical in increasing crystal thickness. To prepare derivative crystals, ligands were individually added to the reservoir solution to a concentration of 5–200 mm. Native crystals were then soaked in the solution for 2–45 min. Scheme B crystals were then transferred into a cryoprotectant solution consisting of the reservoir solution, 100–200 mm of the same ligand, and 20% (v/v) ethylene glycol. Additional cryoprotectant was not necessary for scheme A crystals.

##### X-ray Diffraction, Data Collection and Processing

Crystallographic data were collected at various locations (A, Beamline ID23-2 European Synchrotron Radiation Facility and in-house Rigaku 007HFM rotating anode x-ray generator with a Saturn 944+ CCD detector; B, Beamline I04, Diamond Light Source synchrotron). Data collected in-house were reduced using the HKL2000 package (35). Synchrotron data were processed by the xia2 automated reduction system (36) which makes use of Mosflm (37), Pointless (38), CCP4 (39), and XDS (40). Structures were solved by PHASER molecular replacement (41) using the *S. pneumoniae* NanB structure (PDB code 2VW2 (24)) as the search model. The initial models were autobuilt by ARP/wARP (42) and improved by iterative cycles of manual rebuilding in Coot (43) and refinement in Refmac5 (44) (A and B) and Buster-TNT (45) (B). The PDB_REDO (46) and MolProbity (47) servers were used to inform the refinement and for structure validation. Statistics for data collection and refinement are listed in Table 1.

**TABLE 1:** **Data collection and refinement statistics. Values in parentheses refer to the highest resolution shell**

Crystal	a	b	c	d	e	f	g	h	i
Protein Data Bank code	4YZ1	4YW2	4YZ5	4YW1	4YZ2	4YW3	4YZ4	4YW0	4YW5
CBM complex		6′SL	3′SL	Neu5Ac		Neu5Ac	Neu5Ac	2,3F-Neu5Ac	
Active site complex			Neu5Ac2en	Neu5Ac2en	Neu5Ac2en	Neu5Ac2en		3F-β-Neu5Ac	OC

**Soaking conditions**									
Compound	None	6′SL	3′SL	3′SL	Neu5Ac2en	Neu5Ac2en	Neu5Ac	2,3F-Neu5Ac	OC
Concentration (mm)		20	200	20	50	20	200	5	20
Time (min)		45	2	15	2	45	2	45	5

**Data Collection**									
X-ray source	DLS I04	Rigaku 007HFM	DLS I04	Rigaku 007HFM	DLS I04	Rigaku 007HFM	DLS I04	Rigaku 007HFM	Rigaku 007HFM
Space group and cell dimensions (Å,°)	P2_1_2_1_2_1_	P12_1_1	P2_1_2_1_2_1_	P12_1_1	P2_1_2_1_2_1_	P12_1_1	P2_1_2_1_2_1_	P12_1_1	P12_1_1
	*a* = 150.13, *b* = 73.74, *c* = 136.13	*a* = 100.65, *b* = 74.91, *c* = 113.00, β = 96.21	*a* = 73.47, *b* = 136.57, *c* = 150.07	*a* = 100.83, *b* = 74.95, *c* = 113.30, β = 96.35	*a* = 73.30, *b* = 136.71, *c* = 150.79	*a* = 100.62, *b* = 74.73, *c* = 113.01, β = 96.35	*a* = 73.38, *b* = 136.09, *c* = 150.44,	*a* = 100.24, *b* = 74.84, *c* = 113.35, β = 96.31	*a* = 99.55, *b* = 74.05, *c* = 112.12, β = 95.50
Resolution (Å)	101.05–1.97 (2.2–1.97)	30.00–2.00 (2.05–2.00)	75.04–2.27 (2.33–2.27)	40.00–2.25 (2.31–2.25)	101.28–2.06 (2.11–2.06)	30.00–2.05 (2.10–2.05)	100.92–2.05 (2.21–2.15)	30.00–2.05 (2.10–2.05)	40.00–2.30 (2.34–2.30)
Unique reflections	107,569	109,231	70,273	75,606	93,784	97,981	81,664	102,205	71,545
Completeness (%)	98.52 (98.14)	96.43 (92.05)	99.46 (99.42)	94.15 (95.58)	99.34 (99.33)	93.27 (56.66)	98.77 (97.28)	97.73 (76.33)	99.8 (98.8)
Redundancy	3.1 (3.0)	3.7 (3.6)	4.5 (4.6)	2.6 (2.5)	4 (4)	3.4 (2.2)	5.4 (5.5)	3.5 (2.8)	3.1 (2.8)
*R*_merge_	0.099 (0.533)	0.109 (0.476)	0.326 (1.375)	0.121 (0.482)	0.137 (0.509)	0.096 (0.404)	0.140 (0.636)	0.107 (0.576)	0.089 (0.724)
*I*/σ*I*	13.9 (1.93)	18.39 (3.88)	6.0 (2.5)	11.64 (2.49)	7.1 (2.3)	17.67 (3.02)	7.6 (2.7)	15.97 (2.49)	14.78 (1.93)

**Refinement**									
Reflections used	100,672	103,624	66,621	71,657	89,017	93,033	77,515	96,897	67,786
No. of protein atoms	10,433	10,460	10,503	10,444	10,465	10,463	10,466	10,460	10,452
No. of ligand atoms	60	105	50	84	50	85	60	92	98
No. of water	818	870	721	857	907	728	640	557	344
*R* factor	0.181	0.180	0.218	0.209	0.207	0.172	0.203	0.197	0.207
*R*_free_	0.215	0.209	0.269	0.249	0.243	0.203	0.239	0.233	0.239
Root mean square deviation bond lengths (Å)	0.015	0.012	0.010	0.009	0.017	0.013	0.013	0.016	0.010
Root mean square deviation bond angles (°)	1.643	1.499	1.466	1.396	1.727	1.553	1.537	1.645	1.160
Ramachandran (favored/outlier)	97.02/0.00	97.09/0.00	96.63/0.00	97.03/0.23	96.64/0.00	96.73/0.08	96.94/0.08	97.19/0.08	97.26/0.15
**Average B-factors (Å^2^)**									
All atoms	31.3	25.9	21.5	33.0	15.9	25.3	26.6	31.6	40.9
Protein atoms	31.0	22.7	21.2	33.0	15.3	25.2	26.3	31.7	47.5
Ligand/ion atoms	58.8	35.9	46.5	32.1	39.6	26.7	57.0	29.9	40.6
Waters	32.5	26.9	20.7	33.8	19.8	27.2	28.0	28.8	37.5

##### Isothermal Titration Calorimetry

The NanC CBM was subcloned into the pEHISTEV vector using primers GGCGCCATGGCTCAGGAGACTGAAACTTCTG and TGGTGCTCGAGTTTACATCTTTTTAACAGTTTCTTC and purified according to scheme A with the addition of His_6_ tag cleavage using TEV protease and reverse His tag purification (48). ITC experiments were performed using a VP-ITC microcalorimeter (MicroCal Inc.) with a cell volume of 1.54 ml. Prior to titration, protein samples were exhaustively dialyzed into 20 mm Tris, pH 8, 50 mm NaCl. The ligand was dissolved in the dialysis buffer. Analysis was performed using nonlinear regression and a single binding site model within MicroCal Origin software (version 7.083). Heat of dilution experiments were performed, and the resulting isotherm was subtracted from the experimental conditions.

## Results

### 

#### 

##### Overall Structure

**FIGURE 1.**
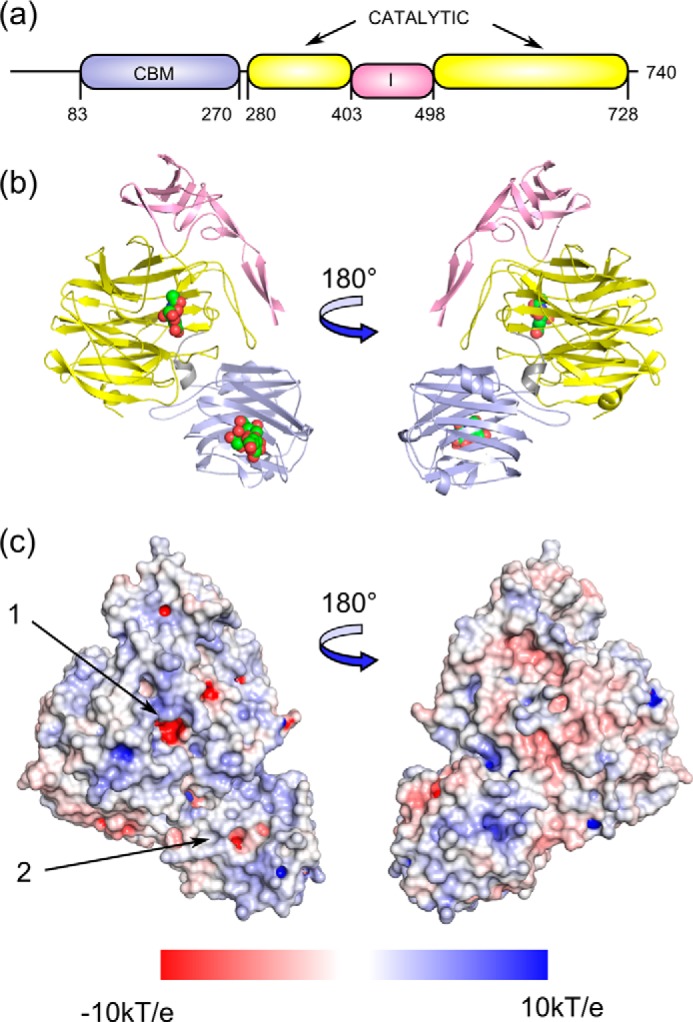
*a*, NanC domain organization and the amino acid boundaries are shown, with the CBM in *light blue*, the sialidase catalytic domain in *yellow*, and the inserted domain in *pink. b*, in the cartoon representation the bound ligands, Neu5Ac2en in the catalytic domain, and 3′SL in the CBM (PDB code 4YZ5) are shown as *spheres. c*, an electrostatic surface has been applied to the unbound crystal structure (PDB code 4YZ1) with the catalytic domain active site and the CBM binding site indicated with *arrows 1* and *2*, respectively. Electrostatic potentials (units of *kT*/*e* from −10 to 10) were calculated by APBS (76) and visualized on the solvent-accessible surface by the program PyMOL (77).

NanC adopts the same overall topology as seen in NanA and NanB, with a CBM (residues 85–271), followed by a catalytic domain (residues 272–740; Fig. 1*a*). The CBM presents a CBM family 40 (CBM40) β-sandwich fold (49) with six-antiparallel strands forming a convex surface and five strands forming a concave, carbohydrate-binding side. The catalytic domain adopts the canonical glycoside hydrolase family 33 sialidase, six-bladed β-propeller fold (Fig. 1*b*) shared by viral, bacterial, and mammalian sialidases. Asp boxes, motifs typical of bacterial sialidases (50), are present in four of the β-propeller blades. Protruding from the catalytic domain is an I domain comprised mainly of β-strands (residues 403–498). I domains are present in the other pneumococcal sialidases (23–25, 51), as well as NanI from *Clostridium perfringens* (52), NanL from *Macrobdella decora* (53), and NanH from *Ruminococcus gnavus* (54). However, I domains are not a general feature of sialidases; for example, they are absent from VcNA from *V. cholerae* (55), Neu2 from *Homo sapiens* (56), and NedA from *Monospora viridifaciens* (57). The function of I domains remains unknown. The absent N-terminal residues include a signal sequence (residues 1–27) that was deleted to aid structural studies and a short region of unknown function (residues 28–84) of which electron density was only observed for residues 83 and 84.

##### NanC-CBM Complexes

**FIGURE 2.**
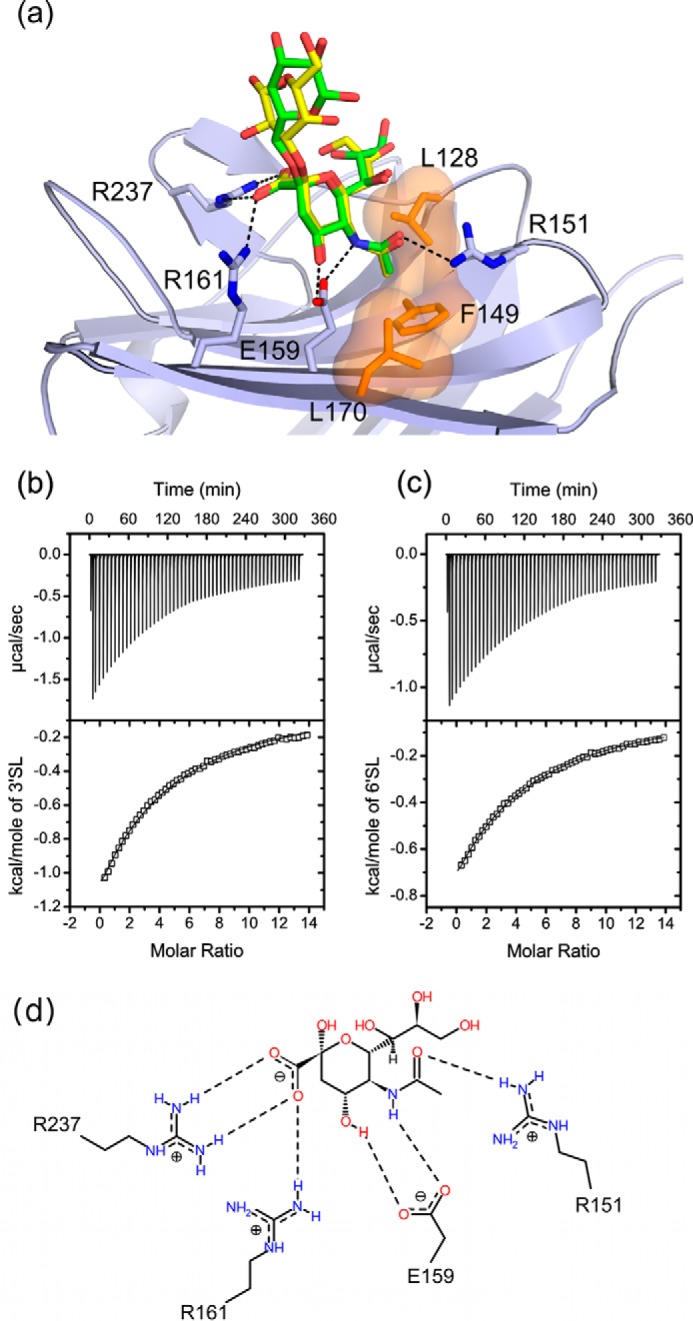
*a*, the CBM binding site of NanC (*light blue*) with superimposed bound 3′SL (*yellow*; PDB code 4YZ5) and 6′SL (*green*; PDB code 4YW2) molecules shown with selected interacting residues. *Dashed black lines* indicate hydrogen-bonding interactions and a semitransparent surface (*orange*) applied to Leu-128, Phe-149, and Leu-170 indicates the hydrophobic pocket. *b* and *c*, ITC experiments assessing the binding of 3′SL and 6′SL, respectively, to subcloned NanC CBM. Based on crystallographic evidence, the binding curve was calculated using a fixed substrate to binding site stoichiometry of 1:1. For 3′SL binding constant *K* = 677 ± 7.44 m^−1^, Δ*H* = −11.4 ± 0.078 kcal/mol, *T*Δ*S* = −7.54 kcal/mol corresponding to a *K_d_* of 1.48 mm. χ^2^/DoF = 92.49. For 6′SL binding constant *K* = 626 ± 4.43 m^−1^, Δ*H* = 8.09 ± 0.036 kcal/mol, *T*Δ*S* = −4.26 kcal/mol corresponding to a *K_d_* of 1.60 mm. χ^2^/DoF = 16.78. *d*, a schematic representation of the interactions between Neu5Ac and the CBM binding site (PDB code 4YW1) visualized using Poseview (78).

**FIGURE 3.**
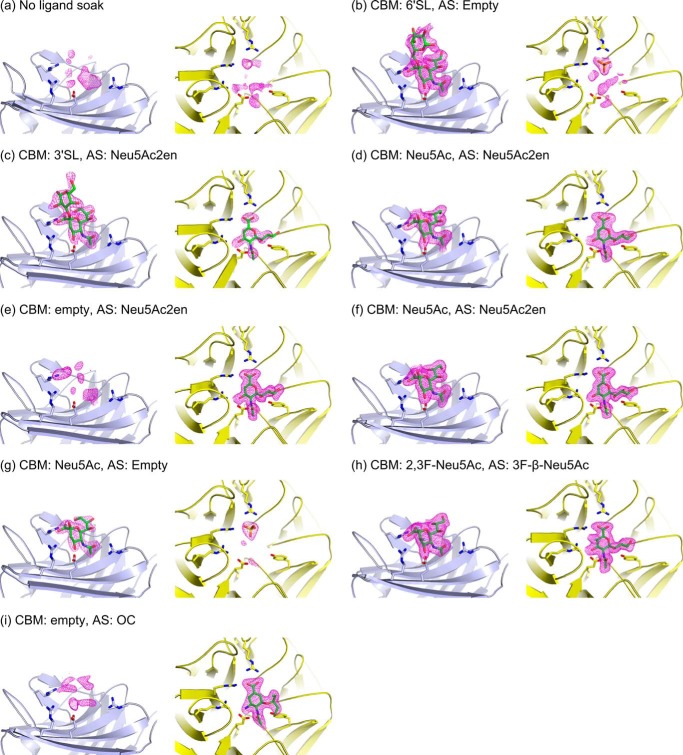
**Views of the CBM binding site (*light blue*) and catalytic domain active site (*AS*, *yellow*) for all described crystal structures.** Bound ligands (*green*) are indicated for both sites. Soaking ligands were: none PDB code 4YZ1 (*a*), 6′SL PDB code 4YW2 (*b*), 3′SL (200 mm for 2 min) PDB code 4YZ5 (*c*), 3′SL (20 mm for 15 min) PDB code 4YW1 (*d*), Neu5Ac2en (50 mm for 2 min) PDB code 4YZ2 (*e*), Neu5Ac2en (20 mm for 45 min) PDB code 4YW3 (*f*), Neu5Ac PDB code 4YZ4 (*g*), 2,3F-Neu5Ac PDB code 4YW0 (*h*), and OC PDB code 4YW5 (*i*). The *F*_o_ − *F*_c_ omit map electron density is shown carved around the ligands and contoured at a level of 3 σ (*magenta mesh*). *Panels a–i* correspond to crystal structures a–i in Table 1.

Structures of the CBM have been obtained in complex with Neu5Ac, 3′SL, and 6′SL (Figs. 2*a* and 3, *a–i*) Neu5Ac complexes were achieved by soaking NanC crystals with Neu5Ac and also by extended soaks (>20 min) with Neu5Ac2en, thus confirming that NanC can hydrate Neu5Ac2en to Neu5Ac. Following short soaks (2 min) with 3′SL and soaks with 6′SL, electron density was observed for the sialyl and galactosyl rings; the glucosyl rings were not visible in electron density. Key interacting residues of the CBM binding site are Arg-237 and Arg-161, which provide electrostatic interactions with the Neu5Ac carboxylate group; Glu-159, which hydrogen bonds to the O4 hydroxyl group; and Arg-151, which hydrogen bonds to the carbonyl of the N-acetyl group (Figs. 2, *a* and *d*, and 3, *b* and *c*). In addition, Phe-149, Leu-128, and Leu-170 form a hydrophobic pocket that accommodates the terminal methyl of the N-acetyl group. Observed protein to ligand interactions are limited to the terminal Neu5Ac residue. Thus the affinities of 3′SL and 6′SL for the NanC CBM were very similar, with *K_d_* values of 1.48 mm (Fig. 2*b*) and 1.60 mm (Fig. 2*c*), respectively, as measured by isothermal titration calorimetry (ITC). The binding site of the CBM is on the same side as the active site of the catalytic domain and the protein face on which they reside displays an overall positive charge (Fig. 1, *b* and *c*). These two factors combined could have a role in orienting the active site in relation to the negatively charged host-bound substrate.

##### The NanC Active Site

**FIGURE 4.**
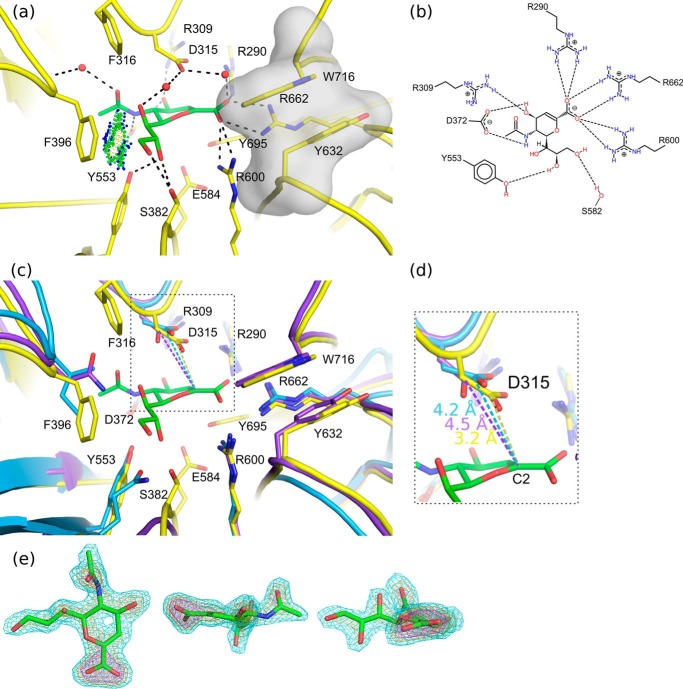
**Neu5Ac2en binding.** *a*, the NanC active site (*yellow*) is shown with Neu5Ac2en (*green*) bound (PDB code 4YW3). Predicted hydrogen bonding interactions are shown with *black dashed lines*. A hydrophobic interaction between the C9 and Phe-396 is indicated with *green* and *blue spheres* (Van der Waals contacts generated using PROBE (79)). The hydrophobic stack providing specificity for α2,3-linked substrates is highlighted with a semitransparent gray surface. *b*, a schematic representation of the interactions between Neu5Ac2en and the active site. *c*, for comparison the active sites of NanA (*blue*) and NanB (*purple*) are aligned onto NanC. *d*, a close-up view of the distances in Å between the Neu5Ac2en anomeric carbon and conserved acid base aspartic acid. *e*, three orientations of the bound Neu5Ac2en ligand are shown. The *F*_o_ − *F*_c_ omit electron density map is contoured at sigma levels of 3 (*cyan*), 5 (*orange*), and 7 (*magenta*) carved at a distance of 1.6 Å.

Structures of NanC have been determined with Neu5Ac2en, 3F-β-Neu5Ac, and OC bound at the catalytic active site (Fig. 3, *c–f*, *h*, and *i*). The NanC active site is a narrow constricted cleft, similar to NanB (24, 25) and contrary to NanA (23, 26). A primary component of this constriction is the hydrophobic stack of Trp-716 and Tyr-632 (Fig. 4*a*). These residues partially block the active site entrance providing specificity for α2–3-linked substrates as opposed to α2–6.

Soaking NanC crystals with 3′SL traps Neu5Ac2en in a half-chair conformation in the active site (Figs. 3, *c* and *d*, and 4*a*). The negatively charged active site shares key features of structurally characterized bacterial, mammalian and parasitic sialidases in general and the pneumococcal sialidases in particular (23–26, 52–59). Thus, many of the Neu5Ac2en to NanC interactions are also conserved with NanA and NanB (Fig. 4, *b* and *c*). These include an arginine triad, comprised of Arg-290, Arg-600, and Arg-662, which orients the ligand in the active site via electrostatic interactions with the Neu5Ac2en carboxylate (Fig. 4, *a* and *b*). The hydrophobic pocket (comprised of Ile-371, Met-390, Phe-316, and Phe-396) accommodates the *N*-acetyl group mirroring the CBM. The ligand sits on top of the catalytic pair of Glu-584 and Tyr-695 and beneath the general acid base Asp-315. The latter residue is substantially closer to the C2 of Neu5Ac2en than in NanA or NanB Neu5Ac2en complexes: 3.2 Å in comparison with 4.2 and 4.5 Å, respectively (Fig. 4*d*; Refs. 23 and 24). Other interacting residues include Ser-582, which is within hydrogen bonding distance of terminal hydroxyls of the Neu5Ac2en glycerol moiety. Arg-309 forms a hydrogen bond with the C4 hydroxyl. Asp-372 forms hydrogen bonds with both the C4 hydroxyl and N5 of the *N*-acetyl moiety.

##### Formation of a Covalent Intermediate in the Active Site

**FIGURE 5.**
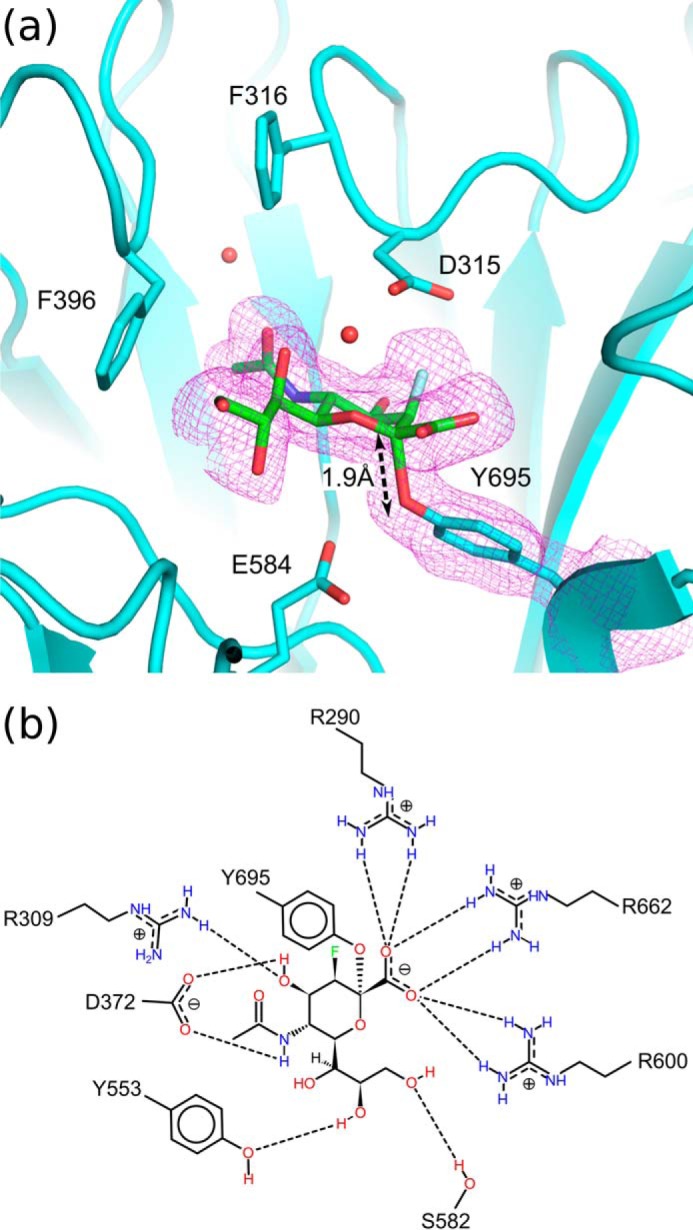
*a*, 3F-β-Neu5Ac covalently bound in the NanC active site (PDB code 4YW0). The *F*_o_ − *F*_c_ omit map electron density map is carved around the ligand and Tyr-695 and contoured at a level of 3 σ (*magenta mesh*). *b*, a schematic representation of the interactions between 3F-β-Neu5Ac and the NanC active site.

NanC crystals soaked with 2,3-difluoro-*N*-acetylneuraminic acid (2,3F-Neu5Ac) presented continuous electron density from the ligand C2 position to Tyr-695 (Fig. 5, *a* and *b*). 3F-β-Neu5Ac was covalently bound to Tyr-695 in an unstrained ^2^C_5_ chair conformation. Equivalent complexes have also been observed in the *C. perfringens* sialidase NanI, the *Trypanosoma rangeli* sialidase TrSA, and the *Trypanosoma cruzi trans-*sialidase TcTS (52, 60, 61). The covalent bond distance is 1.9 Å (Fig. 5*a*). 2,3F-Neu5Ac was also found bound in the CBM binding site (Fig. 3*h*).

##### Plasticity in Active Site 396 Loop

**FIGURE 6.**
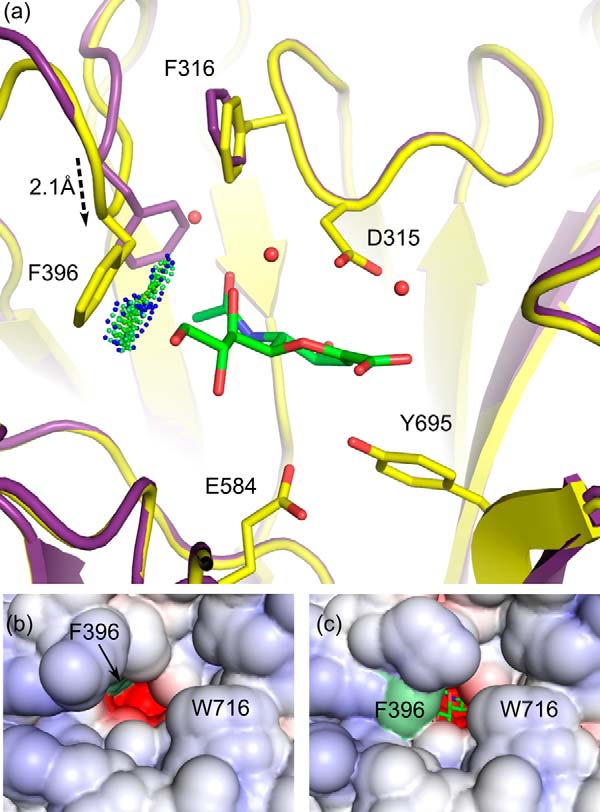
**Structural perturbations in the hydrophobic loop.** *a*, the occluding 396 loop position, when in complex with Neu5Ac2en, is shown in *yellow* (PDB code 4YW0). The nonoccluding loop position, in the unbound active site of the apo-structure, is shown in *purple* (PDB code 4YZ1). The distance between the Phe-396 Cα carbons in the two conformations is 2.1 Å. Neu5Ac2en is shown in *green. Spheres* represent the favorable hydrophobic interactions between Phe-396 and Neu5Ac2en. *b* and *c*, a solvent-accessible surface has been applied to the nonconstricted and constricted conformations, respectively. The position of the Phe-396 residue is highlighted by a *light green surface*.

The most significant structural change observed upon binding and/or formation of Neu5Ac2en is the substantial shift in Phe-316 and Phe-396 of the hydrophobic pocket and the loop upon which the latter resides (herein named the 396 loop, residues 392–401) (Fig. 6*a*). The Cα of Phe-396 advances 2.1 Å into the active site, and the side chain changes conformation, moving out of the active site to accommodate the ligand. The net result of the movement of the 396 loop is that it partially constricts the active site (Fig. 6, *b* and *c*). The movement appears to be stabilized by hydrophobic interactions between Phe-396 and the C9 carbon of Neu5Ac2en because no hydrogen bonds or other stabilizing interactions are formed between the residues of the 396 loop and Neu5Ac2en (Fig. 6*a*).

**FIGURE 7.**
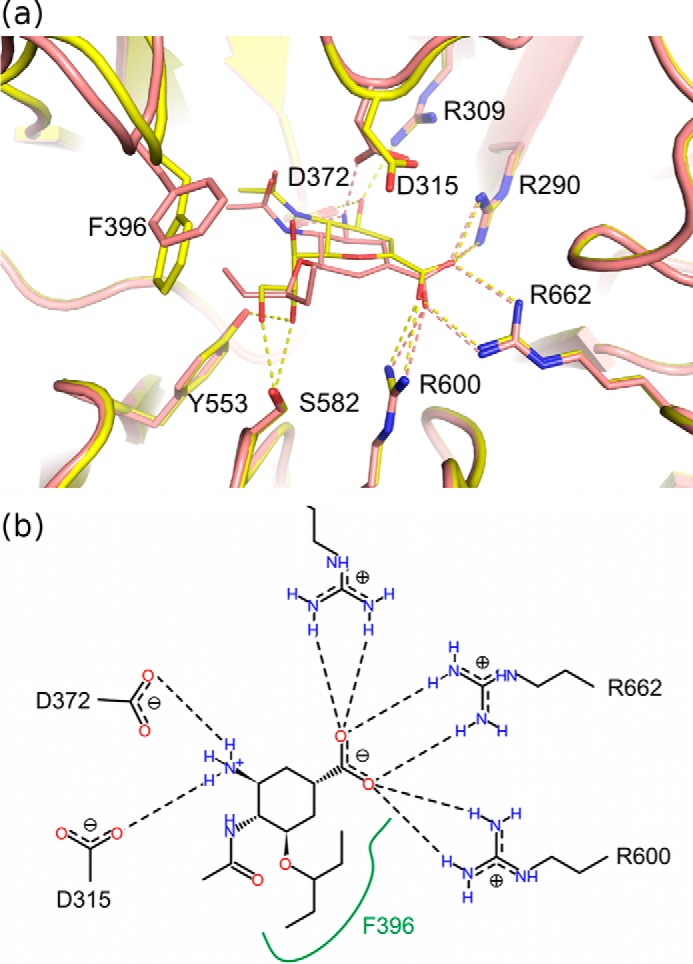
*a*, OC bound in the NanC active site (PDB code 4YW5, *salmon*) in comparison with Neu5Ac2en (PDB code 4YW3, *yellow*). The *dashed lines* in the corresponding color represent hydrogen-bonding interactions. *b*, a schematic representation of the interactions between OC and the NanC active site. The *green line* represents a hydrophobic surface.

Although a poor inhibitor (28), OC binds in the NanC active site (Figs. 3*i* and 7, *a* and *b*). Compared with Neu5Ac2en, OC undergoes a translation of ∼0.8 Å, along a plane approximately defined by the ligand ring, out of the active site. This is likely to be caused by the electrostatic repulsion between the 4-amino group of OC and Arg-309. In comparison with the Neu5Ac2en complex, arginine triad interactions, and hydrogen bonds from Asp-372 to both the 4-amino group and the *N*-acetyl amine group are maintained. The 4-amino group induces a pivot in Asp-315 introducing a new hydrogen-bonding partner. The Neu5Ac2en interactions with Ser-582 and Tyr-553 via the glycerol group are lost. Furthermore, the Neu5Ac2en glycerol group makes favorable hydrophobic interactions with Phe-396 (Fig. 6*a*), whereas the OC pentyl ether group is unable to reproduce this interaction, and the 396 loop does not move into the active site (Fig. 7*a*). This is predicted to be due to steric clashing between Phe-396 and the pentyl group.

## Discussion

NanC is an unusual sialidase in that its primary reaction product is Neu5Ac2en, a nonspecific sialidase inhibitor. We have captured crystal structures of the catalytic site of NanC in complex with its first product, Neu5Ac2en (Fig. 3*d*), and a covalent intermediate using a fluorinated substrate analogue (Fig. 3*h*) and the influenza viral neuraminidase inhibitor OC (Fig. 3*i*). In addition, we have captured complexes of the CBM with Neu5Ac, 3′SL, and 6′SL (Fig. 3, *g*, *c*, and *b*).

### 

#### 

##### The Proposed NanC Reaction Mechanism

**FIGURE 8.**
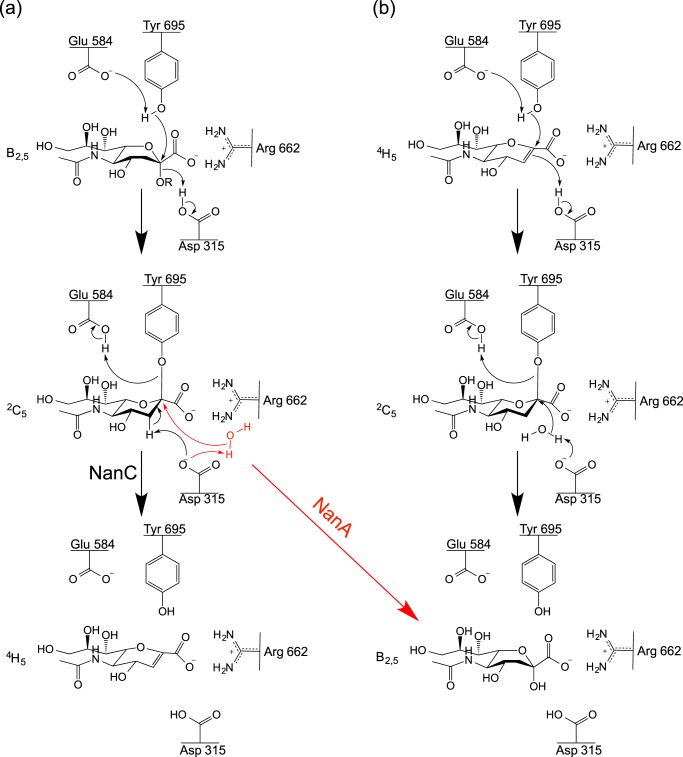
**The proposed NanC reaction mechanism.** *a*, production of Neu5Ac2en from α2,3-linked sialosides. *b*, hydration of Neu5Ac2en to Neu5Ac. As a comparison, the NanA hydrolytic sialidase mechanism is shown in *red* at the point it diverges from the NanC mechanism.

The presented crystal structures provide insight into the NanC reaction specificity and mechanism. Following substrate selection, the substrate is orientated through electrostatic interactions between the Neu5Ac carboxylic acid and the arginine triad. This brings the C2 position into proximity with Tyr-695, which can perform a nucleophilic attack. This step appears to be common to all three pneumococcal sialidases (27). Tyr-695 was confirmed as the nucleophile by soaking a NanC crystal with 2,3F-Neu5Ac, capturing a sialyl-enzyme intermediate with 3F-β-Neu5Ac covalently bound to Tyr-695. The electronegative fluorine atoms at positions C2 and C3 allow trapping of the covalent intermediate by respectively providing a good leaving group and destabilizing the formation of the positive charged transition state required for enzyme desialylation (62). At this point in a classical hydrolytic sialidase, such as NanA, the intermediate would undergo nucleophilic attack by an activated water molecule at the C2 anomeric carbon, releasing Neu5Ac (Fig. 8). However, when NanC crystals were soaked with 3′SL, Neu5Ac2en is found in the active site. Thus we propose that Asp-315 abstracts the proton directly from the C3 position, eliminating Tyr-695 and forming Neu5Ac2en (Fig. 8*a*).

When the pool of substrate is exhausted and Neu5Ac2en is in excess, NanC can hydrate Neu5Ac2en to Neu5Ac. When short 3′SL soaks are performed, electron density is observed for uncleaved 3′SL in the CBM binding site with Neu5Ac2en in the active site (Fig. 3*c*). When longer soaks were performed, Neu5Ac2en is still observed in the active site, and cleaved Neu5Ac was observed in the CBM binding site (Fig. 3*d*). When short Neu5Ac2en soaks were performed, the CBM binding site was empty, whereas bound Neu5Ac became apparent when the soaking time was increased. This is consistent with ^1^H NMR experiments where Neu5Ac2en is formed after 0.5 h and is hydrated to Neu5Ac over a further period of 6 h (27). During Neu5Ac2en hydration to Neu5Ac, there would not be a leaving glycan preventing solvent access to the ligand C2 position, allowing the hydration reaction to proceed via the mechanism described for the *C. perfringens* sialidase NanI by Newstead *et al.* (27, 52). In short, the nucleophilic electrons in the Neu5Ac2en π-bond attack the nearby Asp-315 proton. This generates a delocalized positive charge at the ligand C2 position, stabilized by Tyr-695 and vulnerable to nucleophilic attack by an activated water molecule, resulting in the formation of Neu5Ac (Fig. 8*b*).

##### Reaction Determinants

The NanC active site is similar to that of other sialidases. However, there are important differences. First, Asp-315 is ∼1 Å closer to the C2 of Neu5Ac2en than the equivalent residue in NanA and NanB (Fig. 4*d*). This advanced position would allow the C3 proton to be abstracted directly. In NanA and NanB, there is space for attack at the C2 position by an activated water molecule forming Neu5Ac, and the C6 glycerol moiety forming 2,7-anhydro-Neu5Ac, respectively, which is not the case in NanC. Mutation of Asp-315 to Ala abolishes catalytic activity (27).

The data presented here also highlight plasticity in the hydrophobic 396 loop. Upon binding to Neu5Ac2en, Phe-396, at the loop apex, is brought into proximity of the ligand by loop movement (Fig. 6*a*). This conformational change is a key feature because the movement constricts the opening to the active site in a manner that would make it almost watertight (Fig. 6*c*). Mutating NanC Phe-396 to the hydrophilic Asn, as is found in NanB, leads to the production of a mixture of both Neu5Ac2en and Neu5Ac (27). The Trp-716 hydrophobic stack and the leaving aglycone group would further exclude water from the active site. This desolvated environment would promote attack by Asp-315 on the C3 proton. Striking active site loop movements have also been observed in the influenza virus neuraminidases (63) and the human sialidase Neu2 (56).

##### Enzyme Specificity

The affinities of the NanC CBM for 3′SL and 6′SL are similar, at 1.48 and 1.60 mm, respectively (Fig. 2, *b* and *c*). The *V. cholerae* sialidase also has a sialic acid binding CBM; however, the affinities for 3′SL and 6′SL are much tighter, at 18 and 19 μm, respectively (64). The *V. cholerae* CBM is proposed to be part of a second CBM40 subfamily (49). Low affinity could imply that NanC occupies a substrate-rich environment. Analogously, the tight affinity of the *V. cholerae* CBM40 may suggest a requirement to withstand forces in the gut that are absent in the NanC niche. In contrast to the less specific CBM, strict active site specificity for the α2,3-glycosidic linkage is achieved via constriction of the entrance by a hydrophobic stack of Trp-716 and Tyr-632 (Fig. 4*a*). This has also been seen in the intramolecular *trans*-sialidases NanB (24, 25) and *M. decora* NanL (53). In the human respiratory system, α2,3-glycosidic linkage preference would make NanC more active in the lungs rather than the upper respiratory tract, which primarily expresses α2–6-linked sialosides (65). Contrasting specificity profiles are also observed for the characterized human sialidases, with the most broadly and highly expressed sialidase, Neu1, presenting the widest substrate range (66).

NanC encoding pneumococcal strains are over-represented in invasive isolates from human cerebral spinal fluid in comparison with noninvasive isolates from the nasopharynx (21). The CNS is rich in NanC substrate molecules with α2–3-linked sialoglycans found throughout, with a multitude of functions (67). Parker *et al.* (68) identified contributions to NanC reaction selectivity from components of the leaving group. In particular, NanC cleaved α2–3-linked sialyl-LacNAc more effectively than α2–3-linked sialyl-lactose. α2,3-Linked sialyl-LacNAc is present in GD1a and GT1b gangliosides, which account for 25 and 18% of human brain gangliosides, respectively. Residues in the vicinity of the leaving group that could contribute to this selectivity include Arg-397, Arg-718, and Trp-716. Finally, NanC does not cleave *N*-glycolylneuraminic acid containing sialosides (68). Like in the CBM binding site, the active site holds the ligand *N*-acetyl group in a hydrophobic pocket, which would preclude accommodation of Gc-containing ligands. Humans have lost the ability to synthesize *N*-glycolylneuraminic acid, although it is expressed in many non-human mammals including non-human hominids such as chimpanzees, bonobos, gorillas, and orangutans (69). Dietary *N*-glycolylneuraminic acid can be incorporated into human glycoproteins (70); however, it does not access the CNS (71). Although it has been linked to haemolytic-uraemic syndrome (22), the role of NanC in pneumococcal disease is as yet not understood. The over-representation of NanC in invasive isolates and CNS isolates and adaptation to ligands abundant in the CNS suggest that it may have an important role in pathogenesis of invasive pneumococcal disease such as bacterial meningitis.

##### Oseltamivir Carboxylate Binding

OC is an antiviral agent designed to target the influenza virus neuraminidase. It has also demonstrated efficacy against *S. pneumoniae* infections of the respiratory tract (72, 73). NanA is inhibited by OC with a *K_i_* of 1.77 μm, whereas no effective inhibition is observed against NanB or NanC (28). The manner of OC to NanC binding is similar to that of the previously solved NanA OC complex (51). In particular, the OC 4-amino group forms hydrogen bonds with Asp-372, and repulsion by Arg-351 causes a shift out of the active site. In the NanA complex, the OC pentyl ether group forms hydrophobic interactions with Leu-583 and Ile-427 (51). Conversely, in NanC the pentyl ether group prevents the 396 loop adopting the constricted conformation, because of a steric clash (Fig. 7*a*). In comparison with NanA and NanC, the equivalent position in NanB or Neu2 is hydrophilic (24, 25, 56). Correspondingly, OC is a very poor inhibitor of Neu2 (*K_i_* greater than 6 mm). This is in stark contrast to the low micromolar inhibition observed for Neu5Ac2en and zanamivir, which can contribute numerous hydrogen bonding interactions to the pocket via the C6 glycerol groups (31, 56, 74) (PDB ID: 2F0Z). These variations in protein-ligand interactions highlight the difficulties in translating structure based drug developments between sialidases, because although the active sites are often similar, their ability to bind individual inhibitors differs substantially.

##### Conclusions

The results described here present a structurally rationalized and novel mechanism for the unusual production of Neu5Ac2en by the NanC sialidase and completes the structural description of pneumococcal sialidase catalysis. The mechanistic diversity among the three sialidases, NanA, NanB, and NanC, is achieved through subtle amino acid substitutions, positional shifts of conserved residues, and active site plasticity; the active site core is otherwise preserved. These changes hint at the evolutionary pressure to adapt to environmental niches, which vary in substrate type and quantity and underline the importance of the sialidases for bacterial nutrition and successful host cell invasion. In summary, these works highlight the importance of thorough structural characterization of sialidases; although they may appear, superficially, very similar, they show significant mechanistic diversity.

## Author Contributions

G. L. T. and M. A. W. conceived and supervised the work. C. D. O. and P. L. separately expressed, crystallized, and solved the crystal structure of NanC according to schemes A and B, respectively. C. D. O. performed the ITC analysis. O. S. and M. A. W. performed initial crystallographic analysis. J. A. P., G. L. T., and M. A. W. contributed to data analysis. C. D. O. and P. L. led writing of the paper with contributions from J. A. P., G. L. T., and M. A. W. All authors analyzed the results and approved the final version of the manuscript.

## Footnotes

5 The abbreviations used are:

CBM: carbohydrate-binding module
Neu5Ac2en: 2-deoxy-2,3-didehydro-*N*-acetylneuraminic acid
Neu5Ac: *N*-acetylneuraminic acid
3F-β-Neu5Ac: 3-fluoro-β-*N*-acetylneuraminic acid
OC: oseltamivir carboxylate
3′SL: α2–3-sialyllactose
6′SL: α2–6-sialyllactose
PDB: Protein Data Bank
ITC: isothermal titration calorimetry
2,3F-Neu5Ac: 2,3-difluoro-*N*-acetylneuraminic acid.
